# T-2 Toxin Induces Apoptotic Cell Death and Protective Autophagy in Mouse Microglia BV2 Cells

**DOI:** 10.3390/jof8080761

**Published:** 2022-07-22

**Authors:** Tun Sun, Qinzhi Zhang, Meng Li, Shusheng Tang, Chongshan Dai

**Affiliations:** 1 College of Veterinary Medicine, China Agricultural University, No. 2 Yuanmingyuan West Road, Beijing 100193, China; suntun@cau.edu.cn (T.S.); 2016305010216@cau.edu.cn (Q.Z.); s20193050723@cau.edu.cn (M.L.); 2 Beijing Key Laboratory of Detection Technology for Animal-Derived Food Safety, Beijing 100193, China; 3 Key Biology Laboratory of Chinese Veterinary Medicine, Ministry of Agriculture and Rural Affairs, Beijing 100193, China

**Keywords:** T-2 toxin, oxidative stress, mitochondrial apoptotic pathway, autophagy, Nrf2/HO-1 pathway

## Abstract

T-2 toxin exposure could cause neurotoxicity; however, the precise molecular mechanisms remain unclear. In the present study, we investigated T-2 toxin-induced cytotoxicity and underlying molecular mechanisms using a mouse microglia BV2 cell line. The results show that T-2 toxin treatment-induced cytotoxicity of BV2 cells was dose- and time-dependent. Compared to the control, T-2 toxin treatment at 1.25–5 ng/mL significantly increased reactive oxygen species (ROS) production and triggered oxidative stress. T-2 toxin treatment also caused mitochondrial dysfunction in BV2 cells, which was evidenced by decreased mitochondrial transmembrane potential, upregulated expression of Bax protein, and decreased expression of Bcl-2 protein. Meanwhile, T-2 toxin treatment upregulated the expression of cleaved-caspase-3, cleaved-PARP-1 proteins, and downregulated the expression of HO-1 and nuclear Nrf2 proteins, finally inducing cell apoptosis in BV2 cells. N-acetylcysteine (NAC) supplementation significantly attenuated T-2 toxin-induced cytotoxicity. Moreover, T-2 toxin treatment activated autophagy and upregulated autophagy flux, and the inhibition of autophagy significantly promoted T-2 toxin-induced cell apoptosis. Taken together, our results reveal that T-2 toxin-induced cytotoxicity in BV2 cells involves the production of ROS, the activation of the mitochondrial apoptotic pathway, and the inhibition of the Nrf2/HO-1 pathway. Our study offers new insight into the underlying molecular mechanisms in T-2 toxin-mediated neurotoxicity.

## 1. Introduction

**Figure 1:**
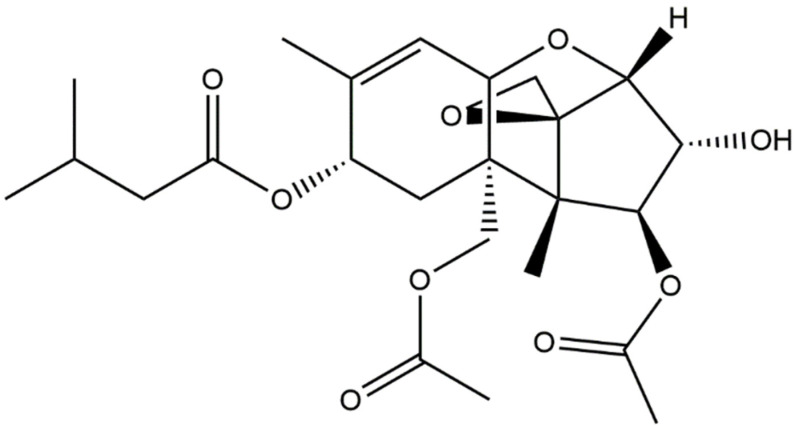
The structure of T-2 toxin.

T-2 toxin (Figure 1), an inevitable environmental pollutant, is the most toxic type A trichothecene mycotoxin and could cause various health problems in human and animals [1]. T-2 toxin is a natural toxin and is produced by various *Fusarium* species, including *F. acuminatum*, *F. equiseti*, *F. sporotichioides*, and *F. poae*. T-2 toxin is highly stable and resistant to various environmental factors, including heat and ultraviolet light [2]. It has been reported that T-2 toxin was detected in corn, wheat, barley, and rice crops in the field, during storage, or in drinking water in several provinces of China (e.g., Qinghai and Sichuan Provinces) [3]. T-2 toxin was detected in Chinese herbs [4,5]. It has been demonstrated that T-2 toxin can be easily absorbed through gut and pulmonary mucosa and the skin system, damaging multiple organs of animals and humans, including skin, kidneys, liver, brain, heart, spleen, the hematopoietic system, the lymphoid system, gastrointestinal bone marrow, and the reproductive system [6,7,8,9,10,11,12,13,14,15,16,17,18,19,20,21]. T-2 toxin contamination poses a potential public health risk.

Recent evidence indicated that T-2 toxin could cross the blood–brain barrier, accumulate in brain tissues, and cause neurotoxicity [22,23,24]. In mouse or rat models, T-2 toxin exposure has resulted in various neurological symptoms, including muscular weakness, depression, and ataxia anorectic responses [25,26]. Some studies found that T-2 toxin-induced neurotoxicity involved the production of reactive oxygen species (ROS), oxidative stress, mitochondrial dysfunction, cell cycle arrest, DNA damage, inflammatory response, the nuclear factor erythroid 2-related factor (Nrf2)/heme oxygenase-1 (HO-1) pathway, the nuclear factor kappa light chain enhancer of activated B cells (NF-κB) pathway, the cyclic-AMP response binding protein (CREB) pathway, the mitogen-activated protein kinase (MAPK) pathway, and the HMGB1 pathway [1,27,28,29,30]. It is urgent to understand the precise molecular mechanisms, which could support more effective strategies to improve and prevent T-2 toxin-induced neurotoxicity in clinical settings. For example, several studies found that curcumin supplementation, a natural antioxidant, could significantly inhibit renal oxidative stress damage in rats or chicken caused by other mycotoxins (i.e., ochratoxin A and aflatoxin B1) [31,32].

In recent years, the functions of glial cells, namely, astrocytes and microglia, have been noted in various neurodegenerative diseases [33,34,35]. They play important roles in maintaining brain homeostasis and regulating neuroinflammation, neural information transmission, and neuronal cell survival [33,34,35]. It has been demonstrated that some environmental toxins, including polybrominated diphenyl ethers quinone, cadmium, and methylmercury, can induce cell apoptosis and neuroinflammation in astrocytes and microglia, and play a critical role in the development of toxin-induced neurotoxicity or neurodegenerative diseases [36,37,38]. A previous study showed that T-2 toxin exposure could induce marked cytotoxicity in human astrocytes [24]. T-2 toxin could be rapidly metabolized to HT-2 toxin, T-2-triol, T-2-tetraol, 3-hydroxy-T-2, and 3-hydroxy-HT-2 toxin in liver via the CYP450 metabolic enzymes [39]. It has been demonstrated that T-2 toxin is more toxic than these main metabolites [39,40]. A previous study reported that the metabolization of T-2 toxin in human astrocytes is slower than that in human colon carcinoma HT-29 cell, and consistently increased toxicity sensitivity in human astrocytes was detected [24]. Such evidence indicates that T-2 toxin exhibits various effects in different cell types. To date, there is little information about the toxic mechanism of T-2 toxin in microglia cells. Therefore, in the present study, we investigated the cytotoxicity of T-2 toxin on mouse microglia BV2 cells. Furthermore, the underlying molecular mechanisms were explored. 

## 2. Materials and Methods

### 2.1. Chemicals and Reagents

T-2 toxin (CAS NO. 21259-20-1) (purity ≥ 99%) was purchased from Sigma-Aldrich (St. Louis, MO, USA). Sodium dodecyl sulfonate (SDS), dimethyl sulfoxide (DMSO), chloroquine phosphate salt (CQ) (purity ≥ 99%), and Tris hydroxymethyl (Tris-HCl) were purchased from AMRESCO Inc. (Solon, OH, USA). Rhodamine (Rh) 123, 0.05% Trypsin-EDTA, phenylmethylsulfonyl fluoride (PMSF), 1% (*v*/*v*) penicillin and streptomycin, N-acetylcysteine (NAC), and 2′,7′-dichlorfluorescein-diacetate (DCFH-DA) were purchased from Beyotime (Shanghai, China). T-2 toxin was prepared in DMSO at a concentration of 10 μg/mL stock solution and stored at −20 °C. All other reagents were of the highest analytical grade available.

### 2.2. Cell Culture

Mouse BV2 microglia were purchased from China Center for Type Culture Collection (Wuhan, China). Cells were cultured in DMEM medium (Gibco, Grand Island, NY, USA) complemented with 10% (*v*/*v*) fetal bovine serum (FBS) (Gibco BRL, Grand Island, NY, USA), 1% penicillin, and streptomycin in cell incubator at 37 °C and 5% CO_2_.

### 2.3. Measurement of Cell Viability

The cell activities were measured using the CCK-8 Kit according to our previous study [1]. In brief, BV2 cells were seeded into 96-well plates at the density of 1 × 10^4^ cells/well. After 12 h, cells were treated with T-2 toxin at various concentrations from 0.625 to 10 ng/mL for 6, 12, and 24 h. In the control group, cells were treated with the equal vehicle (i.e., 0.1% DMSO). To identify the roles of oxidative stress and autophagy in T-2 toxin-induced cytotoxicity, cells were pretreated with NAC at 2.5 mM or CQ at 5 μM for 1 h, followed by co-treatment with T-2 toxin at 2.5 ng/mL for additional 24 h. After treatments, cells were added to 10 μL CCK-8 solution and incubated at 37 °C for 1 h. The cells in the control group were treated with an equal volume of vehicle (i.e., 0.1% DMSO). The absorbance at 450 nm was recorded by a microplate reader (Tecan Trading AG, Männedorf, Switzerland). All experiments were independently repeated three times.

### 2.4. Measurement of Apoptosis

Cell apoptosis rates were measured by using the Annexin V-FITC apoptosis detection kit (Vazyme, Nanjing, China) with flow cytometer analysis, according to our previous report [41]. Briefly, BV2 cells were seeded at the density of 4 × 10^5^ cells/well into 6-well plates. After 12 h, cells were exposed to T-2 toxin at doses of 1.25, 2.5, and 5 ng/mL for 24 h. In the control group, cells were treated with an equal volume of vehicle (i.e., 0.1% DMSO). Then, cells were washed with PBS twice, trypsinized with no- EDTA, and finally harvested in a 1.5 mL sterile microcentrifuge tube. Then, cells were resuspended in 500 μL Annexin V-FITC binding buffer and stained with 5 μL Annexin V-FITC solution and 5 μL PI solution from kits. After 15 min incubation in the dark at room temperature, the rates of apoptosis were analyzed by flow cytometry (Becton Dickinson, San Jose, CA, USA). All measurements were independently performed three times.

### 2.5. Measurement of ROS Production and Biomarkers of Oxidative Stress

The production of intracellular ROS was examined by using the DCFH-DA staining method, according to the previous studies [1,41]. In brief, BV2 cells were seeded at the density of 4 × 10^5^ cells/well into 6-well plates. After 12 h, cells were treated with T-2 toxin at 1.25, 2.5, and 5 ng/mL for 24 h. Then, cells were cultured with fresh medium containing 10 μg/mL DCFH-DA for 30 min at 37 °C in the dark. In the control group, cells were treated with an equal volume of vehicle (i.e., 0.1% DMSO). Finally, cells were washed with PBS twice and observed by a fluorescent microscope (excitation wavelength: 488 nm; emission wavelength: 525 nm) (Leica Microsystems, Wetzlar, Germany). Fifty cells in each group were randomly selected, and the values of fluorescence intensity were quantitatively analyzed using ImageJ software. We also assessed the effects of NAC treatment on T-2 toxin-induced ROS production. In brief, cells were treated with NAC at 2.5 mM for 2 h, then co-treated with or without T-2 toxin at 2.5 ng/mL for 24 h. Intracellular ROS levels were detected using flow cytometer.

After T-2 toxin treatments, the biomarkers of oxidative stress, including the levels of malondialdehyde (MDA) and the activities of catalase (CAT) and dismutase (SOD), were measured according to the manufacturer’s instructions for the MDA, CAT, and SOD commercial kits (Nanjing Jiancheng Biological Engineering, Nanjing, China). The protein concentration of each sample was determined by BCA assay kit (Thermo Fisher Scientific Inc., Waltham, MA, USA). The detail protocols were performed according to the manufacturer’s instructions. Finally, MDA levels, CAT activities, and SOD activities of each sample were normalized to corresponding protein concentrations.

### 2.6. Measurement of Caspase-3 Activities

The activities of caspase-3 were measured by a commercial caspase-3 activity kit, according to the manufacturer’s instructions. In brief, cells were pretreated with NAC at 10 mM or CQ at 5 μM for 1 h, followed by treatment with T-2 toxin at 2.5 ng/mL for an additional 24 h. In the control group, cells were treated with the equal vehicle (i.e., 0.1% DMSO). After treatment, cells were harvested and caspase-3 activities were measured. The protein concentration of each sample was determined by a BCA assay kit (Thermo Fisher Scientific Inc., Waltham, MA, USA). The values were normalized to the control group and results are shown as the fold change in control.

### 2.7. Mitochondrial Membrane Potential Measurement

The changes in mitochondrial membrane potential were measured by using the Rh123 method, according to our previous study [42]. In brief, BV2 cells were seeded at the density of 4 × 10^5^ cells/well into 6-well plates. After 16 h, cells were treated with T-2 toxin at 1.25, 2.5, and 5 ng/mL for 24 h. Then, cells were cultured with fresh medium containing 1 μg/mL Rh123 for 30 min at 37 °C in the dark. In the control group, cells were treated with an equal volume of vehicle (i.e., 0.1% DMSO). Finally, cells were washed with PBS twice and observed by using a fluorescent microscope (excitation wavelength: 488 nm; emission wavelength: 525 nm) (Leica Microsystems, Wetzlar, Germany). Fifty cells in each group were randomly selected, and the values of fluorescence intensity were quantitatively analyzed using ImageJ software.

### 2.8. mRFP-GFP-LC3 Plasmid Transfection

BV2 cells were transiently transfected with the mRFP-GFP-LC3 vector, according to our published study [43]. In brief, BV2 cells were transfected with LC3-RFP-GFP plasmids using X-treme GENE HP DNA transfection reagents (Roche, Basel, Switzerland) for 24 h, then treated with either CQ at 5 μM, T-2 toxin at 2.5 ng/mL, or co-treatment for 12 h. In the control group, cells were treated with an equal volume of vehicle (i.e., 0.1% DMSO). LC3 puncta was observed under LSM 510 Meta Confocal Microscope (Carl Zeiss Micro Imaging, Breda, The Netherlands).

### 2.9. Western Blotting

After treatments, BV2 cells were washed with PBS. Cells were lysed by RIPA lysis buffer (No. P0013B, Beyotime, Haimen, China) with a protease inhibitor cocktail (1 μg/mL leupeptin, 1 μg/mL pepstatin A, 1 μg/mL aprotinin, and 1 mM PMSF) for 15 min at 4 °C. Then, cell samples were ultrasonicated (3 s apart in each cycle for 5 times, power 22 W) using an Ultrasonic Processor (Branson, MO, USA). They were centrifuged at 14,000× *g* for 15 min at 4 °C, and the supernatants were collected. The nuclear protein was exacted using the Nuclear and Cytoplasmic Protein Extraction Kit (Beyotime, Haimen, China), according to the manufacturer’s protocol. The protein concentrations were qualified by using a BCA protein assay kit (Thermo Fisher Scientific Inc., Waltham, MA, USA). Western blotting was performed according to our previously published protocols [44,45].

In brief, 15 μg of the total protein was separated by 8–12% SDS-polyacrylamide gel electrophoresis (PAGE) and transferred by polyvinylidene fluoride (PVDF) membrane. The primary antibodies used were rabbit monoclonal antibodies against poly (ADP-ribose) polymerase-1 (PARP-1), Bcl-2, caspase-3, Histon H3, Nrf2, LC3I/II, and HO-1 (1:1000; Proteintech, Chicago, IL, USA), β-actin (1:1000; Cell Signaling Technology, Danvers, MA, USA), and mouse monoclonal Bax (1:1000; Cell Signaling Technology, Danvers, MA, USA). Secondary antibodies (1:5000, Cell Signaling Technology, Danvers, MA, USA) against mouse or rabbit were incubated for 2 h at room temperature. The relative levels of each protein expression were examined by using a chemiluminescent (ECL) gel imaging system (Tanon, Shanghai, China) and analyzed using ImageJ software (NIH, Bethesda, MD, USA). The levels of these targeted proteins are normalized to β-actin.

### 2.10. Statistical Analysis

All results of current study are presented as mean ± standard deviation (SD), unless otherwise noted. Data analysis was performed by one-way analysis of variance (ANOVA) with Tukey’s post-hoc test using GraphPad Prism 9.0 software (Graph Pad Software, Inc., San Diego, CA, USA). A *p*-value less than 0.05 was considered as statistically significant.

## 3. Results

### 3.1. Cytotoxic Effect of T-2 Toxin in BV2 Cells

**Figure 2:**
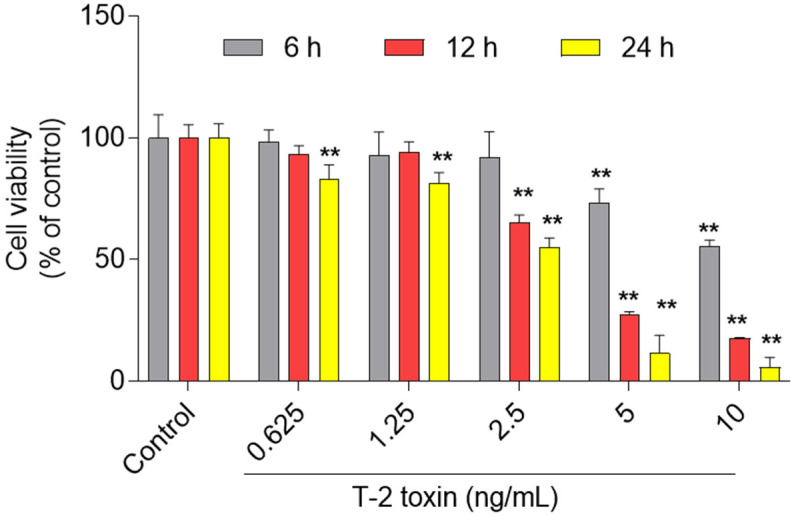
Cytotoxic effects of T-2 toxin on BV2 cells. BV2 cells were treated with T-2 toxin at the final doses of 0.625, 1.25, 2.5, 5, and 10 ng/mL for 6, 12, and 24 h, respectively; cell viabilities were measured. All data are shown as the mean ± SD from three independent experiments (*n* = 3). ** *p* < 0.01, compared to the control group.

As shown in Figure 2, T-2 toxin treatment at the dose range of 0.625–10 ng/mL for 6–24 h could induce marked cytotoxicity and was dose- and time-dependent. At 6 h, T-2 toxin treatment at 0.625, 1.25, 2.5, 5, and 10 ng/mL decreased the cell activities to 98.2%, 92.7%, 92.0%, 73.2% (*p* < 0.01), and 55.7% (*p* < 0.01), respectively; at 24 h, the cell activities decreased to 83.1%, 81.3%, 55.0%, 11.6%, and 5.8%, respectively (all *p* < 0.01).

### 3.2. T-2 Toxin Induces Apoptotic Cell Death in BV2 Cells

**Figure 3:**
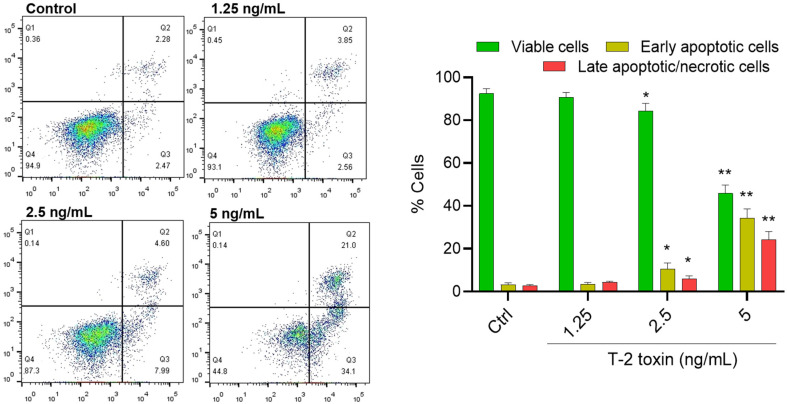
T-2 toxin induces apoptotic cell death in BV2 cells. BV2 cells were treated with T-2 toxin at final concentrations of 1.25, 2.5, and 5 ng/mL for 24 h. Apoptotic rates of T-2 toxin-treated BV2 cells were analyzed by flow cytometry following annexin V-FITV/PI staining. Q1, necrosis cells; Q2, later apoptotic cells; Q3, early apoptotic cells; Q4, live cells. The results are presented as the mean ± SD from three independent experiments (*n* = 3). * *p* < 0.05 and ** *p* < 0.01, compared to the control group.

T-2 toxin treatment at the doses of 0, 1.25, 2.5, and 5 ng/mL for 24 h could induce cell apoptosis in BV2 cells. As shown in Figure 3, compared to the control group, the rates of early apoptotic cells increased from 2.74% to 2.56%, 7.99% (*p* < 0.05), and 34.1% (*p* < 0.01) in BV2 cells treated with T-2 toxin at 0, 1.25, 2.5, and 5 ng/mL, respectively. The rates of late apoptotic cells increased from 2.28% to 3.85%, 4.60% (*p* < 0.05), and 21.0% (*p* < 0.01), respectively, in BV2 cells treated with T-2 toxin at 1.25, 2.5, and 5 ng/mL, respectively.

### 3.3. T-2 Toxin Induces Oxidative Stress in BV2 Cells

**Figure 4:**
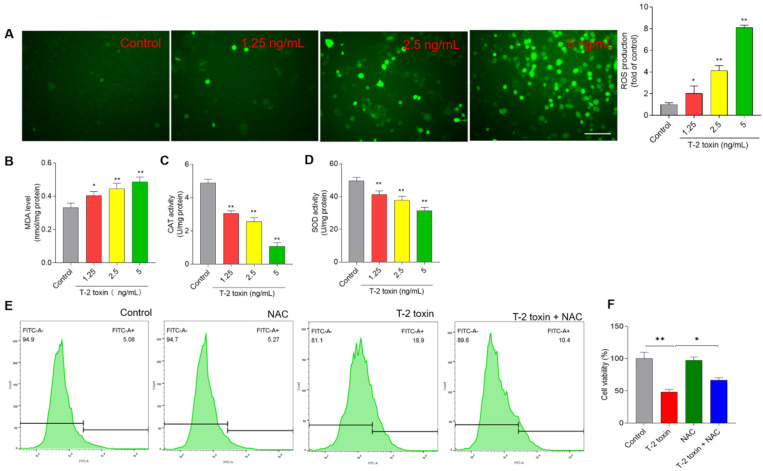
T-2 toxin treatment induces the production of ROS in BV2 cells. (**A**) Cells were treated with T-2 toxin at the final concentrations of 0, 1.25, 2.5, and 5 ng/mL for 24 h; the levels of intracellular ROS were measured by staining with DCFH-DA. The representative images (on the left) were selected (on the left) and quantitative analysis (on the right) was performed. Bar = 20 μm. (**B**) The levels of MDA. (**C**) The levels of CAT activities. (**D**) The levels of SOD activities. (**E**) Cells were pre-treated with NAC at 2.5 mM for 2 h, then co-treated with or without T-2 toxin at 2.5 ng/mL for 24 h; intracellular ROS levels were detected using flow cytometer. (**F**) The effects of NAC pretreatment on T-2 toxin-induced loss of cell viability. Cell treatment is same as in (**E**). All results are presented as mean ± SD from three independent experiments (*n* = 3). * *p* < 0.05 and ** *p* < 0.01, compared to the control.

Compared to the control group, T-2 toxin treatment at 1.25, 2.5, and 5 ng/mL for 24 h significantly increased the levels of ROS 2.1- (*p* < 0.05), 4.1- (*p* < 0.01), and 8.2-fold (*p* < 0.01), respectively (Figure 4A). Furthermore, the biomarkers of oxidative stress, including levels of MDA and activities of SOD and CAT, were measured in BV2 cells. T-2 toxin treatment at 1.25, 2.5, and 5 ng/mL for 24 h significantly increased the levels of MDA to 0.40 nmol/mg protein, 0.44 nmol/mg protein, and 0.49 nmol/mg protein (all *p* < 0.05 or 0.01) (Figure 4B), respectively; decreased the activities of CAT to 3.1 U/mg protein, 2.6 U/mg protein, and 1.1 U/mg protein (Figure 4C) (all *p* < 0.05 or 0.01), respectively; and decreased activities of SOD to 41.3 U/mg protein, 37.7 U/mg protein, and 32.3 U/mg protein (all *p* < 0.01) (Figure 4D), respectively. To confirm the role of oxidative stress in T-2 toxin-induced apoptosis in BV2 cells, the effects of antioxidant supplementation (i.e., NAC) on T-2 toxin-induced ROS production and cytotoxicity were performed. As shown in Figure 4E, NAC pretreatment inhibited the T-2 toxin-induced production of ROS, and attenuated the T-2 toxin-induced decrease in cell viabilities (Figure 4F), compared to the treatment group with T-2 toxin alone.

### 3.4. T-2 Toxin Induces Loss of Mitochondrial Membrane Potential in BV2 Cells

**Figure 5:**
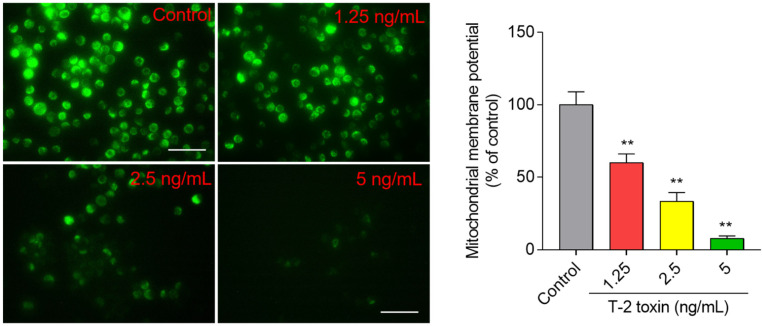
T-2 toxin induces loss of mitochondrial membrane potential in BV2 cells. Cells were treated with T-2 toxin at the final concentrations of 1.25, 2.5, and 5 ng/mL for 24 h, then stained with Rh123 and observed by a fluorescence microscope. The representative images (on the left) were selected and quantitative analysis (on the right) was performed. Bar = 20 μm. Results are presented as mean ± SD from three independent experiments (*n* = 3). ** *p* < 0.01, compared to the control.

Compared to the untreated control cells, T-2 toxin treatment induced decreases in mitochondrial membrane potential (MMP) in a dose- and time-dependent manner. At final concentrations of 1.25, 2.5, and 5 ng/mL for 24 h, the mitochondrial membrane potential (MMP) significantly decreased to 55.9%, 31.1%, and 7.1% (all *p* < 0.05 or 0.01) (Figure 5), respectively.

### 3.5. T-2 Toxin Treatment Activates Mitochondrial Apoptosis Pathway and Downregulated Nrf2/HO-1 Pathway in BV2 Cells

**Figure 6:**
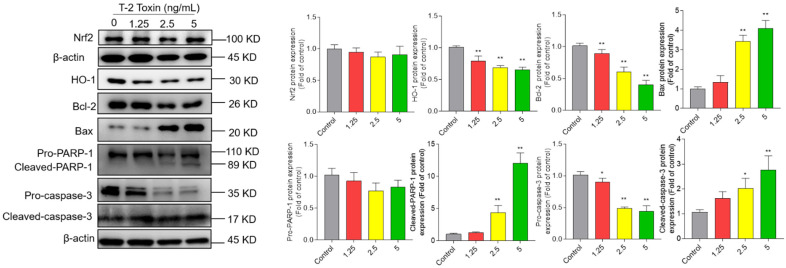
Effects of T-2 toxin on the expression of Nrf2, HO-1, Bcl-2, Bax, cleaved PARP-1, and cleaved caspase-3 proteins. The representative images (on the left) were selected and quantitative analysis (on the right) was performed. Results are presented as mean ± SD from three independent experiments (*n* = 3). * *p* < 0.05 and ** *p* < 0.01, compared to the control.

As shown in Figure 6, T-2 toxin treatment significantly increased the expressions of Bax, cleaved-PARP-1, and cleaved-caspase-3 proteins and decreased the expressions of pro-caspase-3, HO-1, and Bcl-2 proteins. Compared to the control group, T-2 toxin treatment at 5 ng/mL significantly decreased the expression of HO-1 to 0.65-fold (*p* < 0.01) and increased the expression of cleaved PARP-1, cleaved caspase-3 proteins, and the ratio of Bax/Bcl-2 to 12.1-, 2.8-, and 10.2-fold (all *p* < 0.01), respectively. T-2 toxin treatment slightly decreased the expression of total Nrf2 protein, but there were no significant changes compared to the control group. However, we further found that T-2 toxin treatment at 2.5 and 5 ng/mL could significantly inhibit the expression of nuclear Nrf2 (Supplementary Figure S1).

### 3.6. T-2 Toxin Treatment Activates Cell Autophagy and Autophagy Plays a Protective Role

**Figure 7:**
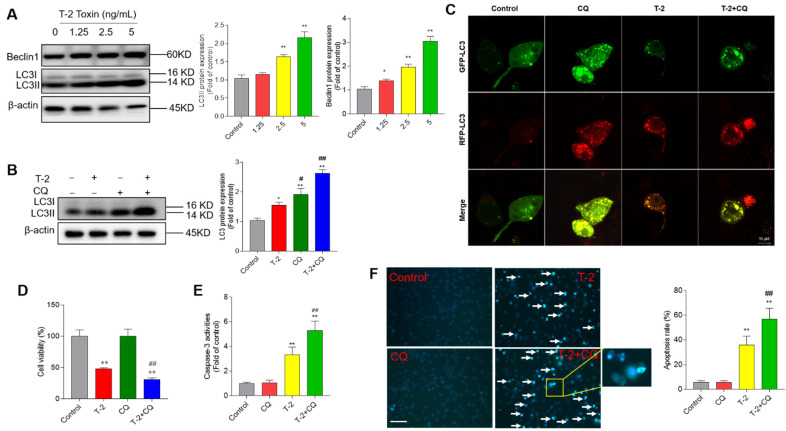
T-2 toxin treatment activates cell autophagy and autophagy play a protective role. (**A**) BV2 cells were treated with T-2 toxin at 1.25, 2.5, and 5 ng/mL for 24 h; the expressions of Beclin1 and LC3II proteins were measured by Western blot. The representative bands (on the left) and quantitative analysis (on the right) are shown. (**B**) BV2 cells were pre-treated with chloroquine (CQ) at 5 μM for 1 h, then co-treated with T-2 toxin at 2.5 ng/mL for an additional 24 h; the expression of LC3II protein was measured. (**C**) Autophagy flux was monitored by the mRFP-GFP-LC3 plasmid transfection method. Cells were treated with Q at 5 μM, T-2 toxin at 2.5 ng/mL, or co-treatment for 12 h; autophagosomes and autolysosomes were observed and photographed by a laser scanning confocal microscope. Bar = 10 μm. (**D**–**F**) Same treatments in BV2 cells as in B; cell viabilities (**D**), caspase-3 activities (**E**), and cell apoptotic rates (**F**) were measured. The results are shown as the means ± SD from three independent experiments (*n* = 3). * *p* < 0.05 and ** *p* < 0.01, compared to the control; ^#^ *p* < 0.05, and ^##^ *p* < 0.01, compared to the T-2 alone treatment group.

As shown in Figure 7A, T-2 toxin treatment significantly increased the expression of Beclin1 and LC3II proteins. When cells were treated with T-2 toxin at 5 ng/mL for 24 h, the expression of LC3II and Beclin1 proteins increased to 2.2- and 3.1-fold, respectively (both *p* < 0.01) (Figure 7A), compared to the corresponding control. Furthermore, our results showed that inhibition of autophagy by CQ further promoted the expression of LC3II compared to treatment with T-2 toxin alone (Figure 7B). Moreover, autophagy flux in T-2 toxin-treated cells was further assessed by using mRFP-GFP-LC3 transfection. As shown in Figure 7C, BV2 cells were treated with T-2 toxin at 2.5 ng/mL for 12 h, and increased formation of autophagosomes and autolysosomes, evidenced by yellow and red puncta, respectively, was detected. Meanwhile, the number of autolysosomes (red puncta) was greater than autophagosomes (yellow puncta), which was partly reversed by CQ cotreatment, which indicates that T-2 toxin treatment activated autophagy and upregulated autophagy flux in BV2 cells. In addition, CQ treatment promoted the T-2 toxin-induced decreases in cell viabilities and increases in caspase-3 activities and apoptotic rates in BV2 cells (Figure 7D–F). Treatment with CQ alone did not affect the cell viabilities, caspase-3 activities, or apoptotic rates of Bv2 cells, compared to the control group.

## 4. Discussion

T-2 toxin is the most toxic type A trichothecene mycotoxin, and can be detected in grain, animal feed, drinking water, and Chinese herbs [2,3,4,5]. It also can accumulate in the body of animals and humans by feed or food consumption and cause potential health risks [2,3,4,5]. A recent study showed that T-2 toxin was detected in 2.3% of human urine samples from 260 rural residents of Nanjing City, China [46]. T-2 toxin could cross the blood–brain barrier and induce neurotoxicity [30]. Guo et al. found that oral administration of T-2 toxin at a signal dose 2 mg/kg could induce marked neurological symptoms and brain pathology injury in rats [47]. Our recent studies showed that T-2 toxin exposure in the range of 5 ng/mL to 80 ng/mL for 24 h could induce apoptotic cell death in N2a and PC12 neuronal cells [1,28]. It has been demonstrated that neural disorders are related to functional or histologic damage to glial cells [48]. Of note, microglial cells act as macrophages in the brain tissues and play a critical role in the maintenance of the microenvironment of brain cells [48]. The activation of microglial cells could induce neuroinflammation and participate in neurodevelopmental dysfunction as well as psychiatric and neurodegenerative disorders [49]. In the present study, our results showed that T-2 toxin in the range of 0.625 ng/mL to 10 ng/mL could induce cytotoxicity in BV2 cells in a dose- and time- dependent manner (Figure 2). These data indicate that microglial cells are highly sensitive to T-2 toxin exposure. It may also play a critical role in T-2 toxin-induced neurotoxicity. For the first time, we found in this current study that T-2 toxin-induced cytotoxicity in BV2 cells involves the production of excessive ROS, oxidative stress, mitochondrial dysfunction, induction of autophagy, and the activation of mitochondrial apoptotic pathway (Figure 3, Figure 4, Figure 5, Figure 6 and Figure 7).

Oxidative stress is defined as the consequence of the imbalance between intracellular ROS production and endogenous antioxidant systems in the process of damage or stress [50]. In general, nerve cells enriched with high polyunsaturated fatty are more vulnerable to oxidative stress-related injury [51]. Excessive ROS production could damage membranes, lipids, proteins, and DNA, resulting in various toxic effects in cells [52]. Like the previous studies [1,27], in the present study, T-2 toxin treatment could significantly elevate the production of ROS (Figure 4). Significantly increased levels of MDA, a biomarker of lipid peroxidation [31,32,42], were also detected in the T-2 toxin-treated BV2 cells (Figure 4). Furthermore, antioxidant NAC supplementation partly inhibited T-2 toxin-induced ROS production, then inhibited T-2 toxin exposure-induced cytotoxicity (Figure 4), indicating that excessive ROS and oxidative stress damage partly contribute to T-2 toxin-induced cell death. In addition, we also found that activities of the endogenous antioxidant enzymes SOD and CAT were significantly inhibited in T-2 toxin-treated cells (Figure 4). The expression of SOD and CAT could be transcriptionally regulated by Nrf2, a housekeeping transcription factor responsible for the regulation of cellular redox balance in mammals [53]. In the previous study, T-2 toxin treatment also significantly inhibited the activities of SOD and CAT with the decreases in HO-1 expression in N2a neuronal cells [1]. In the present study, the total expression of Nrf2 protein after T-2 toxin treatment did not change, but nuclear Nrf2 expression was significantly inhibited (Supplementary Figure S1). As with SOD and CAT, the HO-1 gene could be transcriptionally regulated by Nrf2 and exhibited anti-oxidative stress effects [53]. Taken together, the inhibition of nuclear Nrf2 may contribute to the transcriptional inhibition of SOD, CAT, and HO-1, then exacerbate T-2 toxin-induced cytotoxicity. The precise molecular mechanisms remain to be further elucidated.

Mitochondria are the main productor of ROS, and are also one of its targets. Excessive ROS could directly activate mitochondrial permeability transition, resulting in the loss of mitochondrial membrane potential and mitochondrial dysfunction, finally inducing cell death [54]. Essentially in mammalian cells, the mitochondrial membrane potential generated by proton pumps (mitochondrial complexes I, III, and IV) is an essential component in the process of energy storage during oxidative phosphorylation. Therefore, it is also usually used as an indicative characteristic parameter of mitochondrial dysfunction caused by drugs or toxins [1,28,41,42,44,55]. In another study, T-2 toxin treatment at 10 nM could cause the marked loss of mitochondrial membrane potential in GH3 cells [56]. Similarly, our recent study showed that T-2 toxin treatment at 5 ng/mL could significantly decrease the mitochondrial membrane potential in N2a neuronal cells [1]. In a rat model, T-2 toxin exposure resulted in marked mitochondria damage in the cerebral cortexes [47]. In the present study, the results showed that T-2 toxin treatment at 12.5–5 ng/mL caused the marked loss of mitochondrial membrane potential in BV2 cells (Figure 5). Notably, the dose of T-2 toxin at 1.25 ng/mL decreased the cell viability to about 92.7%, and the corresponding mitochondrial membrane potential decreased to 55.9% (Figure 1 and Figure 5). This indicates that T-2 toxin could result in mitochondrial dysfunction in BV2 cells. As a consequence, mitochondrial dysfunction usually triggers the mitochondrial apoptotic pathway, which finally causes cell apoptosis, a classic programmed cell death [57]. Indeed, apoptotic cell death has been detected in multiple tested cell lines treated with T-2 toxin, including human hepatocytes (L02), murine Leydig cells, mouse N2a neuronal cells, human primary astrocytes, rat PC12 cells, and porcine renal epithelial cells [24,58,59,60]. Not surprisingly, markedly increased apoptotic rates were detected in BV2 cells (Figure 3). Apoptosis could be triggered by extrinsic and intrinsic pathways. The mitochondrial apoptotic pathway is a critical intrinsic pathway in the process of apoptosis [1,30,60]. Our results found that T-2 toxin treatment significantly upregulated the expression of pro-apoptosis protein Bax and downregulated the anti-apoptosis protein Bcl-2 protein (Figure 6). Meanwhile, T-2 toxin treatment also significantly upregulated the activities of caspase-3 and cleaved caspase-3, and PARP-1 was detected in T-2 toxin-treated BV2 cells (Figure 6). These results are consistent with the previous studies [1]. The downregulation of Bcl-2 and upregulation of Bax can cooperatively permeabilize the mitochondrial outer membrane, followed by the release of cytochrome C (CytC), cascading to induce the activation of caspase-9 and -3 [61]. Activated caspase-3 could cleave PARP-1, important in DNA repair, thus promoting apoptosis [62]. Therefore, cleaved caspase-3 and PARP-1 are usually considered as two important markers of apoptosis [1,62], which has been identified in T-2 toxin-induced apoptosis in human primary astrocytes, rat PC12 cells, and mouse N2a neuronal cells [1,24,28]. Taken together, these findings indicate that the activation of mitochondrial apoptotic pathway contributes to T-2 toxin-induced apoptosis in BV2 cells.

It is known that autophagy is a lysosome-dependent intracellular catabolic process, and its activation could provide a protective role in cells faced with stress or damage caused by drugs or toxins [63]. It has been reported that T-2 toxin could upregulate autophagy in the brain tissue of rats and liver tissue of chickens [47,64]. LC3II and Beclin1 are markers of autophagy and they mediate the initiation and closure of the autophagic vesicle, respectively [43,65]. In the present study, our results show that T-2 toxin treatment significantly upregulated the expression of LC3II and Beclin1 proteins (Figure 7A). CQ is an inhibitor of lysosomal activity and blocks autophagosome–lysosome fusion [43]. CQ was added and co-treated with tested compounds, which aimed to monitor autophagy flux—if further increased expression of LCII protein was detected upon CQ co-treatment with test compounds compared to CQ alone, this indicated the activation of autophagy flux [43]. Our results showed that CQ cotreatment with T-2 toxin further upregulated the expression of LC3II (Figure 7B). In addition, the formation of autophagosomes and autolysosomes and increased autophagy flux were also observed by using mRFP-GFP-LC3 transfection (Figure 7C). Therefore, these data indicate that T-2 toxin treatment could significantly upregulate the autophagy flux in BV2 cells. In addition, we further investigated the functional role in T-2 toxin-induced cytotoxicity. We found that autophagy inhibition by CQ further promoted T-2 toxin-induced the decreases in cell viabilities, caspase-3 activation, and apoptosis (Figure 7D–F). Consistently, Yang et al. found that inhibition of autophagy by CQ could promote T-2 toxin-induced apoptosis in mouse primary Leydig cells [66]. Taken together, our results reveal that T-2 toxin could activate autophagy flux in BV2 cells and that autophagy activation plays a protective role.

**Figure 8:**
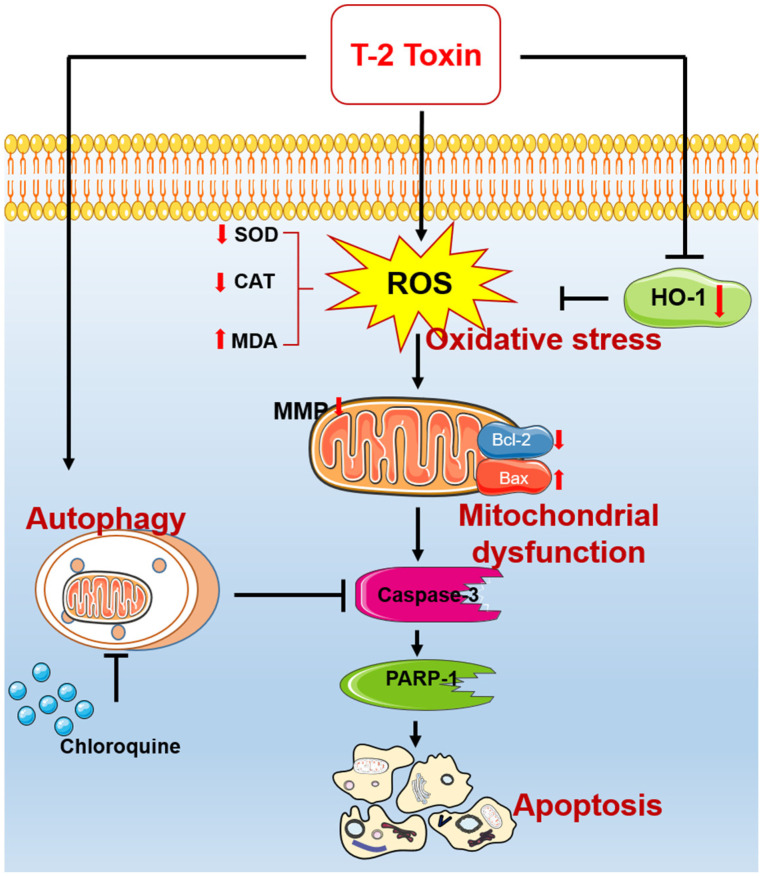
Schematic diagram of the proposed mechanisms of T-2 toxin-induced apoptosis in mouse microglia BV2 cells. ROS, reactive oxygen species; MMP, mitochondrial membrane potential; PARP-1, poly (ADP-ribose) polymerase-1; MDA, malondialdehyde; CAT, catalase; SOD, dismutase; HO-1, heme oxygenase-1.

In conclusion, for the first time, our study reveals that T-2 toxin could promote the production of excessive ROS and inhibit endogenous antioxidant enzyme activities, causing oxidative stress and mitochondrial dysfunction and triggering the mitochondrial apoptotic pathway in mouse microglia BV2 cells (Figure 8). Meanwhile, T-2 toxin could activate autophagy in BV2 cells, and it played a protective role (Figure 8). Our current study provides new insight into understanding the action mechanisms of T-2 toxin neurotoxicity and facilitates the discovery of new treatment strategies.

## Data Availability

Not applicable.

## Supplementary Materials

The following supporting information can be downloaded at: https://www.mdpi.com/article/10.3390/jof8080761/s1, Figure S1: The effect of T-2 treatment on the nuclear Nrf2 protein expression in mouse BV2 cells.
