# Supplementary material for: T-2 Toxin Induces Apoptotic Cell Death and Protective Autophagy in Mouse Microglia BV2 Cells
Source: J Fungi (Basel). 2022 Jul 22;8(8):761. doi: 10.3390/jof8080761 (PMC9330824; doi:10.3390/jof8080761)
Supplement: Supplementary file 1 [file jof-08-00761-s001.zip › jof-1622180-supplementary.pdf]

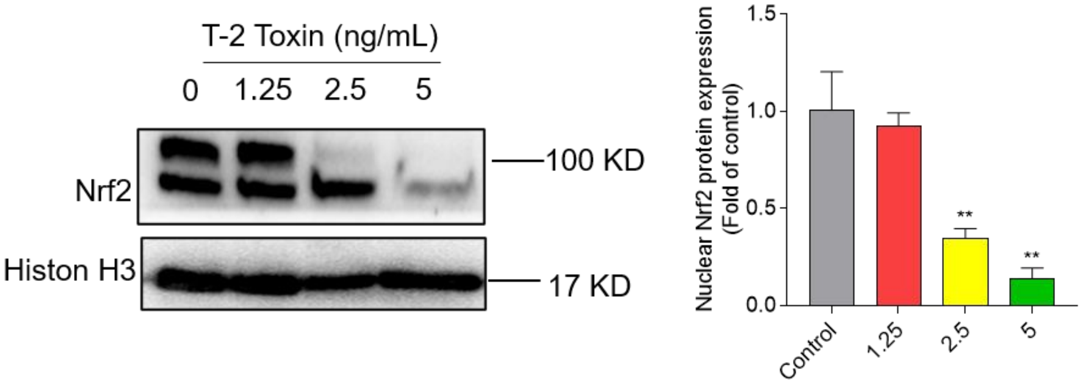

Figure S1: The effect on T-2 treatment on the nuclear Nrf2 protein expression in mouse BV2 cells. The representative images (on the left) were selected and quantitative analysis (on the right) was performed. Results shown present as mean ± SD, from three independent experiments (n=3). * $P < 0.05$, and ** $P < 0.01$, compared to the control.
